# Percutaneous paravalvular leak closure: clinical outcomes and practical insights from a single-center experience in Japan

**DOI:** 10.1007/s12928-026-01273-3

**Published:** 2026-04-24

**Authors:** Go Hashimoto, Kenji Makino, Atsuhiro Saito, Shiho Ide, Sari Waga, Hiromasa Hayama, Norihiro Kogame, Yoshiyuki Yazaki, Hiroki Niikura, Hidehiko Hara

**Affiliations:** ¹Division of Cardiovascular Medicine, Toho University Ohashi Medical Center, Tokyo, Japan

**Keywords:** Paravalvular leak, Transcatheter closure, Prosthetic heart valve, Occlutech PLD, Three-dimensional transesophageal echocardiography (TEE)

## Abstract

While transcatheter paravalvular leak (PVL) closure offers a viable alternative to reoperation in high-risk patients, data from Japan remain limited. To address this, we retrospectively analyzed 28 consecutive patients who underwent percutaneous PVL closure at a single center in Japan between 2014 and 2022 (mitral, *n* = 23, aortic, *n* = 5; median follow-up, 2.0 years). We herein discuss real-time transesophageal echocardiography (TEE) with three-dimensional imaging, guided defect characterization, device sizing, and intra-procedural assessment. The study’s primary clinical endpoint was all-cause mortality, and its primary procedural endpoint was technical success, defined as a residual PVL < moderate on intra-procedural TEE in the absence of emergency surgery or death. The secondary clinical endpoint was a composite of all-cause death, heart failure hospitalization, or reintervention. Technical success was achieved in 71% of patients. Device strategies evolved over time: in earlier cases, the Amplatzer Vascular Plug II (AVP II) and Amplatzer Duct Occluder II (ADO II) were used off-label, whereas later on, dedicated rectangular PVL devices (PLDs) became the preferred choice, typically requiring fewer devices per case. Hemolysis indices (e.g., lactate dehydrogenase and total bilirubin) improved following successful PVL reduction, and patients with a residual PVL < moderate demonstrated superior clinical outcomes at follow-up. To facilitate benchmarking in elderly and renally impaired populations, we collected practical procedural metrics (procedure duration, contrast volume, and radiation exposure). This single-center experience demonstrates that transcatheter PVL closure is both feasible and effective in Japanese practice, while highlighting the importance of device selection and standardized intra-procedural TEE endpoints for procedural success.

**Supplementary Information:**

The online version contains supplementary material available at 10.1007/s12928-026-01273-3.

## Introduction

Cardiac valve replacement can result in postoperative complications that may necessitate reoperation, including thrombosis, prosthetic valve infection, pannus formation, bioprosthetic valve dysfunction, and paravalvular leak (PVL). PVL occurs in 5–18% of all valve replacements (2–10% after aortic valve surgery, 7–17% after mitral valve surgery) [1–5]. Patients with PVL presenting with symptomatic heart failure or hemolytic anemia require invasive intervention.

Transcatheter PVL closure has emerged as a viable treatment option for selected high surgical risk patients. However, this procedure is technically complex, requiring execution by an experienced team of interventional cardiologists, echocardiographers, and anesthesiologists. Accurate preprocedural diagnosis and planning are also critical for success. Previous research indicates that with increasing operator experience and the use of multimodality imaging guidance, procedural success rates can exceed 90%, with low associated complication rates [6–8]. Detailed multimodal imaging, including fluoroscopy, echocardiography, and computed tomography (CT), is essential for improving procedural outcomes.

Device approval and procedural experience in Japan remain limited compared with Europe and the United States. The only case series of PVL closures in Japan is that of Niikura et al., which involved 12 cases treated with the Amplatzer Vascular Plug II (AVP II) [9]. The present report of a comprehensive single-center experience in Japan therefore provides an essential foundation for procedural standardization and future multicenter collaboration.

This study aimed to retrospectively review and present our single-center experience with transcatheter PVL closure, which included both mitral and aortic valves. It represents the largest Japanese cohort to date.

## Methods

### Study population

Between February 1, 2014 and March 4, 2022, a total of 28 patients were clinically evaluated and underwent PVL closure at Toho University Ohashi Medical Center, Tokyo. Patients were considered eligible for percutaneous closure based on the following criteria: (1) dyspnea corresponding to New York Heart Association (NYHA) class III, or class II accompanied by significant lifestyle or occupational impairment or clinically significant hemolytic anemia; (2) moderately severe or severe paravalvular prosthetic regurgitation; (3) absence of active endocarditis; and (4) provision of informed consent to the procedure.

Before providing their informed consent, participants were given detailed information about the risks associated with complex catheter techniques, the off-label use of approved devices, the limited evidence on clinical efficacy, and the available surgical alternatives.

Five of the patients were enrolled from the RESEAL study (tRanscathEter cloSurE of pAravalvular Leaks; UMIN000033433), a prospective multicenter trial in Japan.

Twelve of the patients were treated with AVP II, and overlapped with those previously reported [9], with the present study encompassing all devices and treated patients.

Clinically significant hemolytic anemia was defined as a hemoglobin level < 13.0 g/dL in men or < 12.0 g/dL in women, combined with laboratory evidence of intravascular hemolysis and symptoms severe enough to require blood transfusion.

All cases were reviewed by a multidisciplinary Heart Team comprising interventional cardiologists, cardiac surgeons, and imaging specialists. The team determined the suitability of transcatheter versus surgical reoperation, considering surgical risk, leak morphology, and the likelihood of durable device anchoring. Among the five aortic cases, four involved surgical prosthetic valves and one involved a transcatheter heart valve (Sapien XT 23 mm, Edwards Lifesciences) implanted during a prior TAVR procedure.

The study was approved by the institutional ethics committee **(H24073_H23069_H21012)**, and written informed consent was obtained from all patients in accordance with national ethical guidelines. Patients were also given the opportunity to opt out of participation through public disclosure, as required by the Ethical Guidelines for Medical and Health Research Involving Human Subjects in Japan.

### Percutaneous closure

The 28 patients each underwent a percutaneous PVL closure between February 1, 2014 and March 4, 2022. Five aortic and 23 mitral patients with a total of 67 defects among them were treated using an AVP II, Amplatzer Duct Occluder II (ADO II), or a PVL device (PLD). Procedural metrics, including total procedure duration, contrast volume, and fluoroscopy time, were recorded for all cases. Peak skin dose (in mGy) was also obtained from the catheterization system.

Device selection was based on an assessment of leak morphology, defect size, the risk of prosthetic interference, and sheath compatibility. In the earlier procedures, AVP II and ADO II were used off-label. The dedicated PLD was introduced at our institution through participation in the RESEAL trial and used in selected cases during the study period. When multiple devices were required, a stepwise approach was used, prioritizing the closure of the hemodynamically dominant or largest defect first to minimize any potential interference with the prosthetic leaflets. In one case involving a large mitral PVL with severe left ventricular dysfunction, a transapical approach was successfully employed using rectangular PLDs [10].

Occluder devices (either single or multiple) were deployed under combined fluoroscopic and echocardiographic guidance, taking care to minimize the risk of prosthetic impingement or device embolization. Patients continued their pre-existing antiplatelet and anticoagulant therapy throughout the study period.

Regurgitation severity was assessed before and immediately following the procedure according to the European Society of Cardiology (ESC) and American Society of Echocardiography (ASE) grading guidelines. The assessment used Doppler echocardiography and color flow imaging, and measurements were taken using the GE Vivid E9, Vivid 7, and Vivid E95 systems (GE Healthcare, Boston, USA), as well as the Philips iE33 and EPIQ7 systems (Philips, Eindhoven, Netherlands). Transesophageal echocardiography (TEE), transthoracic echocardiography (TTE), CT, and fluoroscopy were used for procedural assessment, planning, and intraprocedural guidance.

### Clinical follow-up

Patient follow-up involved both telephone interviews and outpatient visits to determine vital status and the occurrence of adverse events. For patients referred from other hospitals, clinical updates provided by their attending physicians were incorporated into the follow-up dataset. The median follow-up duration was 2.0 years (interquartile range [IQR]: 0.7–2.3 years).

Sudden cardiac death was defined as an abrupt and unexpected death, with or without documented ventricular fibrillation, occurring either within 1 h of symptom onset in a previously stable patient or as an unwitnessed nocturnal death without antecedent worsening of symptoms. We also recorded hospitalizations for heart failure, reintervention with transcatheter closure, and surgical repair for PVL recurrence.

### Echocardiography

Echocardiography plays a crucial role in assessing PVL both before and after the procedure. All patients underwent a comprehensive evaluation using both TTE and TEE. These assessments focused on the morphology and function of native and prosthetic valves, as well as the size, location, and shape of each PVL defect.

TEE provided a significant advantage over TTE because it was less susceptible to acoustic shadowing from prosthetic material, which can obscure regurgitation jets and complicate evaluation using TTE. The application of three-dimensional (3D) TEE also further enhanced visualization of PVL morphology, improving accuracy and reproducibility by reducing angle dependency. This technique was also crucial for evaluating residual regurgitant jets, guiding catheter manipulation, facilitating device deployment, confirming device configuration, and monitoring immediate post-procedural outcomes. While 3D TEE requires substantial operator expertise, its superior spatial resolution and reproducibility were clinically significant for increasing procedural success.

Both semi-quantitative and quantitative parameters were employed to grade PVL severity, based on the RESEAL study grading scale and in accordance with the ACC and ESC echocardiography guidelines for prosthetic heart valves [11, 12]. Two experienced echocardiographers independently graded PVL severity using Philips QLAB Cardiac Analysis software. When multiple parameters were available, severity was determined using the concordant classification; in cases of discrepancy, 3D-derived parameters were prioritized.

Inter- and intra-observer variability were evaluated using the intraclass correlation coefficient (ICC) for VC width (3D), VCA (3D), and color flow jet area in 10 randomly selected TEE datasets. The intra-observer ICC values were 0.998 for VC width (3D), 0.999 for VCA (3D), and 0.997 for color flow jet area; inter-observer ICC values were 0.998, 0.997, and 0.997, respectively.

Additional imaging modalities, such as contrast-enhanced CT, were used to evaluate the structural characteristics and the depth of the PVL tract. Post-procedural TEE was performed to assess residual leaks and identify potential complications such as device migration, pericardial effusion, intracardiac thrombus, or new intracardiac shunts. Following the RESEAL criteria, residual PVL severity was adjudicated using maximum vena contracta (VC) width and 3D vena contracta area (VCA) as the primary quantitative parameters.

For mitral PVL, color flow jet area, pulmonary vein flow pattern, and the presence of proximal flow convergence were used as additional supportive parameters. For aortic PVL, the diastolic flow pattern in the descending aorta, presence of proximal flow convergence, and circumferential extent were used as supportive indicators when discrepancies occurred among quantitative measures.

**Fig. 1:**
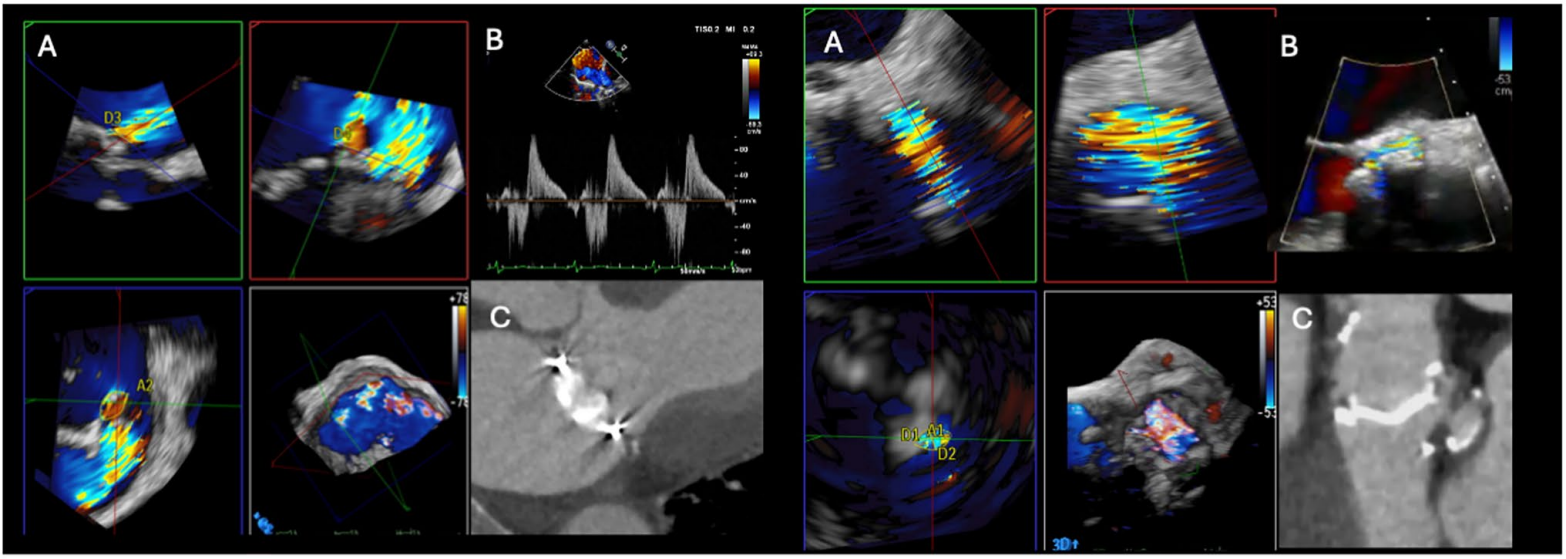
The TEE and CT images for assessing PVL grading. Left: Mitral PVL — (**A**) 3DVCA, (**B**) RUPV systolic flow reversal, (**C**) CT angiography. Right: Aortic PVL — (**A**) 3DVCA, (**B**) 2D long axis PVL flow, (**C**) CT angiography. TEE, transesophageal echocardiography; PVL, paravalvular leak; 3DVCA, three-dimensional vena contracta area; RUPV, right upper pulmonary vein

**Fig. 2:**
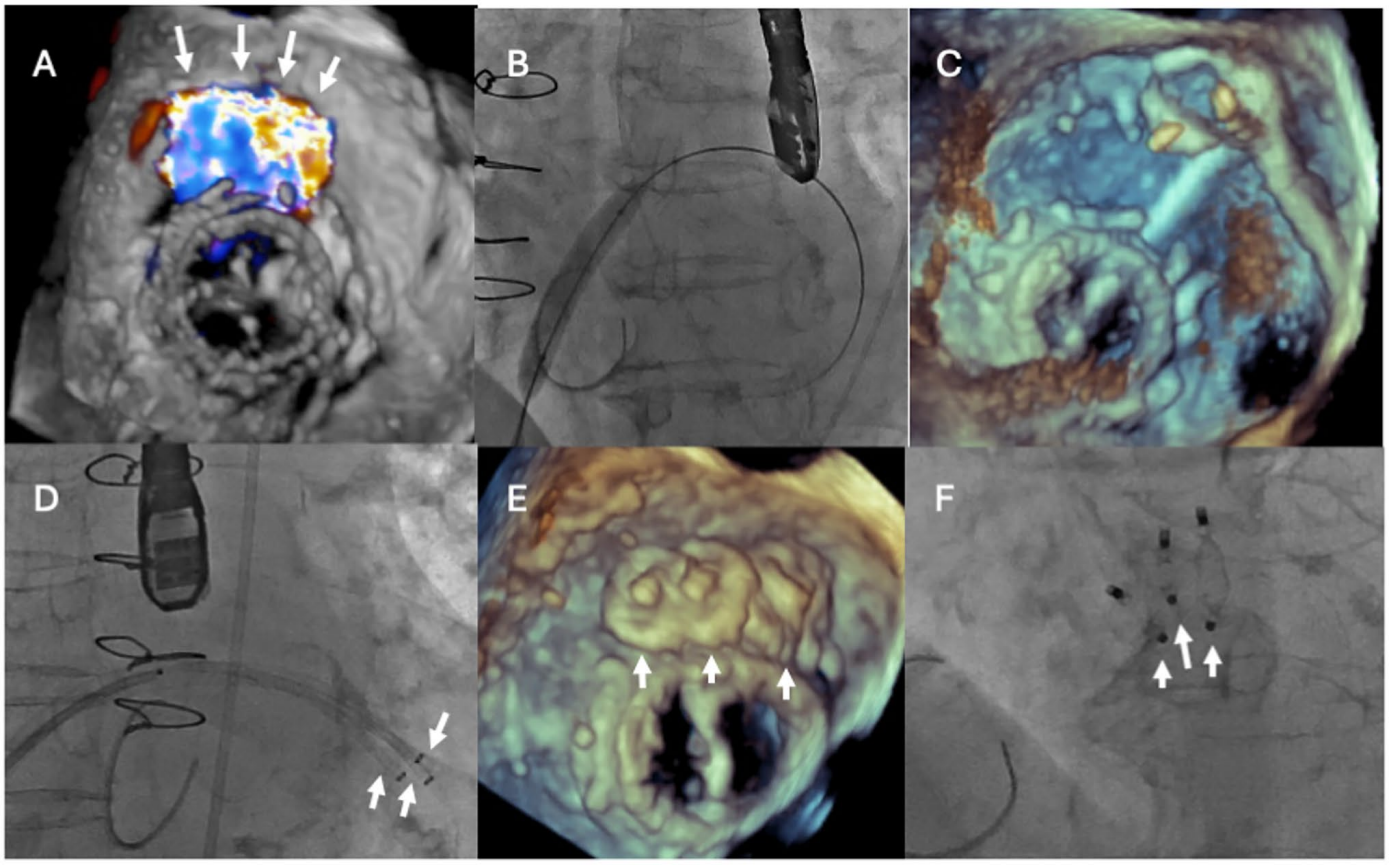
Representative case of anterior mitral paravalvular leak (PVL) closure guided by real-time transesophageal echocardiography (TEE) and fluoroscopy. White arrows indicate the paravalvular leak, delivery sheaths, or deployed occluder devices, as appropriate in each panel. (**A**) Pre-procedural 3D TEE showing a broad anterior mitral PVL (white arrows). (**B**) After transseptal puncture, the atrial septum was dilated with a balloon to facilitate catheter passage (not through the PVL orifice). (**C**) 3D TEE demonstrating advancement of the delivery sheath through the PVL orifice. (**D**) Fluoroscopic image showing three delivery sheaths crossing the defect (white arrows). (**E**) En-face 3D TEE view showing deployment of three Amplatzer Vascular Plug II (AVP II) devices (white arrows) at the anterior aspect of the sewing ring. (**F**) Final fluoroscopic frame confirming stable position of the three devices without interference

Because there is no universally standardized method for quantifying PVL severity, we applied valve-specific grading frameworks. For aortic PVL, we used the Valve Academic Research Consortium-2 (VARC-2) criteria—commonly used for assessing post-transcatheter aortic valve implantation (TAVI) regurgitation—alongside the proposed methodology by Pibarot et al. for prosthetic valve dysfunction [13, 14]. For mitral PVL, we adapted the criteria outlined by Arribas-Jimenez et al. [15], which were originally based on the 2009 ASE prosthetic valve guidelines [11]. These standardized approaches were consistent with those employed in the RESEAL trial (Fig. 1, **Online Resource 1**). Figure 2 illustrates a representative case of anterior mitral PVL closure under real-time 3D TEE and fluoroscopic guidance, demonstrating step-by-step procedural guidance from transseptal puncture and septal dilation to multi-sheath device deployment.

### Data analysis

Depending on data distribution, continuous variables are expressed as the mean ± standard deviation or median (IQR). Categorical variables are reported as counts and percentages. Patient groups—defined by the presence or absence of heart failure (HF) symptoms according to the NYHA functional classification at baseline and after PVL closure—were compared using Student’s *t*-test or the Wilcoxon rank-sum test for continuous variables, and the chi-squared or Fisher’s exact test for categorical variables, as appropriate.

The primary clinical endpoint was all-cause mortality during follow-up. The primary procedural endpoint was technical success, defined as a residual PVL that was less than moderate on intra-procedural TEE in the absence of emergency surgery or death. When echocardiographic parameters were discordant, quantitative three-dimensional parameters, particularly 3D VCA, were prioritized as the primary determinant.

The secondary clinical endpoint was a composite of all-cause death, hospitalization for HF, or reintervention (transcatheter or surgical) for PVL recurrence.

Clinical success was defined as an improvement of at least one NYHA functional class or a significant reduction in hemolytic anemia (reflected by a decrease in serum LDH levels) during follow-up, in the absence of device-related major complications. Procedural and clinical success were assessed independently.

The Kaplan–Meier method was applied to analyze survival and event-free survival, with results presented alongside 95% confidence intervals. All statistical analyses were performed using IBM SPSS Statistics, version 28.0 (IBM Corp., Armonk, NY, USA).

## Results

### Patient characteristics

The cohort comprised 28 patients with a median age of 76 years (IQR 70–80). The baseline clinical characteristics are detailed in Table 1. The primary indication for PVL was most frequently a combination of both HF and hemolytic anemia, observed in 21 patients (75%), representing most of the cohort. The majority of patients (mitral: *n* = 20, 87%, aortic: *n* = 3, 60%) had mechanical valves. Twenty-four patients (86%) had atrial fibrillation, and the median STS score was 10.6% (IQR 6.3–16.9) for mitral and 4.2% (IQR 2.5–14.5) for aortic, suggesting a high risk of in-hospital death following cardiac surgery, especially in mitral patients. Most patients (89%, *n* = 25 out of 28) presented with significant HF symptoms (NYHA class II + III). Cardiac comorbidities were frequent, with hypertension present in 11 (39%) patients and chronic kidney disease in 15 (54%). Table 1 summarizes the medications used for treating these comorbid conditions.

**Table 1:** Baseline clinical characteristics of patients with paravalvular leak

	Mitral (*N* = 23)	Aortic (*N* = 5)
Age, year	76 [71–80]	70 [57–85]
Male	9 (39)	4 (80)
Body surface area, m^2^	1.4 [1.4–1.5]	1.6 [1.5–1.9]
Presenting symptoms Both heart failure and hemolytic anemia Isolated heart failure Isolated hemolytic anemia	18 (78) 2 (9) 3 (13)	3 (60) 2 (40) 0
NYHA classification		
I	2 (7)	0
II	7 (25)	0
III	19 (68)	5 (100)
Systolic blood pressure, mmHg	117 ± 17	120 ± 21
Diastolic blood pressure, mmHg	60 ± 9	56 ± 10
Heart rate, bpm	78 ± 14	71 ± 10
NT-proBNP, pg/ml (*n* = 21)	2096 [1559–3585]	4840 [3313–38332]
Number of sternotomies	1.8 ± 1.0	2.0 ± 1.2
STS predicted risk of mortality, %	10.6 [6.3–16.9]	4.2 [2.5–14.5]
STS predicted risk of morbidity and mortality, %	34.3 [30.8–47.7]	37.1 [25.2–50.1]
Hypertension	9 (39)	2 (40)
Dyslipidemia	3 (13)	1 (20)
Diabetes mellitus	1 (4)	1 (20)
Chronic kidney disease (stage ≥ III)	11 (48)	4 (80)
Coronary artery disease	1 (4)	4 (80)
Stroke	4 (17)	0 (0)
COPD	1 (4)	0 (0)
Mechanical valve Bioprosthetic valve Transcatheter heart valve	20 (87) 3 (13) –	3 (60) 1 (20) 1 (20)
Atrial Fibrillation	21 (91)	3 (60)
Medications		
Warfarin	22 (96)	3 (60)
Aspirin	4 (17)	2 (40)
P2Y12 inhibitor	2 (9)	1 (20)
ACE-I or ARB	6 (26)	2 (40)
Beta-blocker	14 (61)	3 (60)
Calcium channel blocker	4 (17)	1 (20)
Diuretics	19 (83)	5 (100)
Aldosterone antagonist	19 (83)	3 (60)
Digoxin	5 (22)	0

In the aortic cohort, three cases presented with both heart failure and hemolytic anemia. Percentages may not total 100% because of overlapping symptom presentation

One aortic PVL case occurred after TAVR using a Sapien XT 23 mm transcatheter heart valve (Edwards Lifesciences)

Values are shown as number (percentage) or mean ± standard deviation or median [Q1, Q3], as appropriate

NYHA, New York Heart Association Functional Classification; bpm, beats per minute; NT-proBNP, N-terminal pro-brain natriuretic peptide; COPD, chronic obstructive pulmonary disease; ACE-I, angiotensin-converting enzyme; ARB, angiotensin receptor blocker

### Procedural characteristics

A total of 28 percutaneous PVL closure procedures were performed. A transseptal approach was used in 22 (79%) patients, a transaortic retrograde approach in 5 (18%) patients, and a transapical retrograde approach in 1 (4%) patient. The mean number of devices used per procedure was 2.4 ± 1.2 (median 2 [1.25, 3]). The devices included 36 AVP II (54%) and 12 ADO II (18%) devices, as well as 8 PLDs (12%) (Table 2). Procedural success was achieved in 71% of patients (20 of 28).

**Table 2:** Procedural characteristics in patients with mitral and aortic paravalvular leak

*N* = 28	Mitral (*N* = 23)	Aortic (*N* = 5)
Procedure time, min	282 (225, 377)	237 (160, 357)
Contrast volume, mL	5 (0, 20)	3 (0, 13.5)
Fluoroscopy time, min	130 (101, 199)	49.6 (8.5, 152.3)
Peak skin dose, mGy	2050 (1290, 3430)	3570 (1282, 4600)
Length of hospital stay, days	9 (7, 12.5)	7 (7, 7)
Approach		
Antegrade transseptal	22 (79)	0
Retrograde transaortic	0	5 (18)
Retrograde transapical	1 (4)	0
Number of occluder devices per procedure 1 device 2 devices 3 devices 4 devices 5 devices	2.4 ± 1.3 6 (26) 8 (35) 4 (17) 3 (13) 2 (9)	2.2 ± 0.8 1 (20) 2 (40) 2 (40) 0 0
Total number of occluder devices used across all procedures	56	11
Amplatzer vascular plug II	36 (64)	8 (12)
Amplatzer duct occluder II	12 (21)	0
Occlutech paravalvular leak device	8 (14)	3 (4)

Values are shown as number (percentage), mean ± standard deviation, or median [Q1, Q3], as appropriate

The total number of devices exceeds the number of patients because multiple occluder devices were frequently used in a single procedure

PVL, paravalvular leakage; PLD, Occlutech paravalvular leak device

Procedural characteristics are summarized in Table 2. For mitral cases (*n* = 23), the median procedure duration was 282 min (IQR 225–377), the contrast volume was 5 mL (IQR 0–20), the fluoroscopy time was 130 min (IQR 101–199), and the peak skin dose was 2050 mGy (IQR 1290–3430). For aortic cases (*n* = 5), the corresponding values were 237 min (IQR 160–357), 3 mL (IQR 0–13.5), 49.6 min (IQR 8.5–152.3), and 3570 mGy (IQR 1282–4600). Although the peak skin dose was relatively high in some cases, no apparent radiation-induced skin injury was observed during hospitalization or follow-up.

Procedural characteristics according to device type are summarized in Supplementary Table 1. After excluding one case in which both AVP II/ADO II and PLD were used, procedures performed with a dedicated paravalvular leak device required fewer devices per case and were associated with lower radiation exposure compared with those using AVP II/ADO II.

### Post-procedural outcomes

**Fig. 3:**
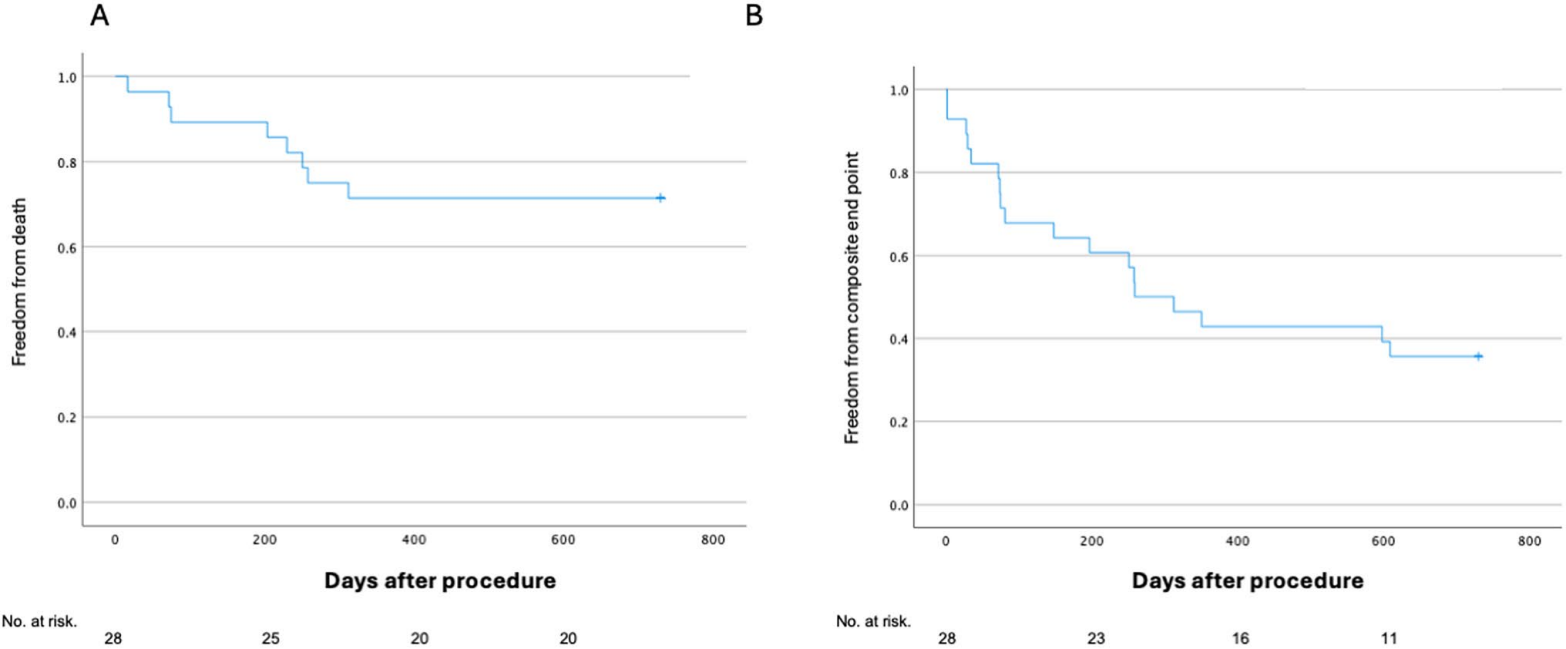
Kaplan–Meier analysis of all-cause mortality and composite clinical outcomes after transcatheter PVL closure. **A**: All-cause mortality observed over 2 years. **B**: Composite outcome (death, heart failure, and re-intervention) over 2 years

During follow-up, the primary clinical endpoint (all-cause mortality) occurred in 8 patients (29%), and the composite endpoint (death, HF hospitalization, or reintervention) occurred in 18 patients (64%) (Fig. 3a, b). There were no in-hospital deaths. Two patients (7%) experienced serious post-procedure complications requiring emergency surgery, including a left ventricular perforation (*n* = 1) during an aortic PVL procedure (post-TAVR) and annulus dehiscence (*n* = 1) during a mitral PVL procedure. Device dislodgement occurred in four cases but was successfully managed by catheter retrieval and reimplantation without further complications.

**Fig. 4:**
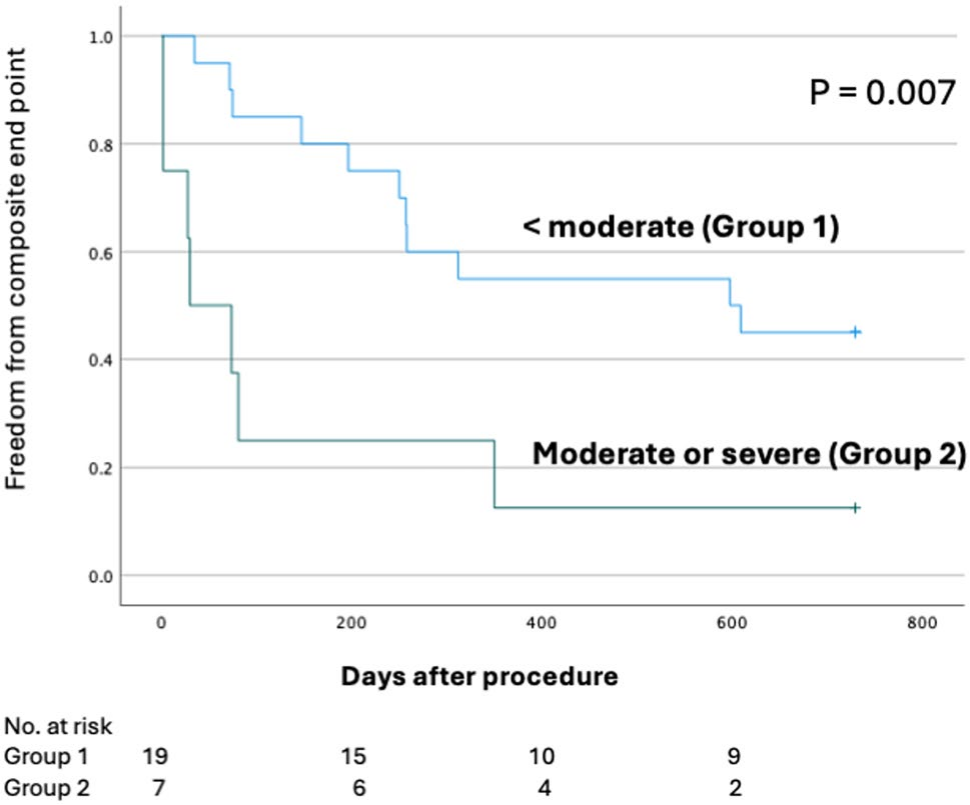
Outcomes of the composite endpoint stratified by PVL severity assessed by intra-procedural transesophageal echocardiography (TEE) at the end of the procedure (< moderate vs. ≥ moderate). Patients with residual PVL < moderate showed significantly better event-free survival than those with PVL ≥ moderate (log-rank *p* = 0.007). PVL, paravalvular leak; TEE, transesophageal echocardiography

Over the two-year follow-up period, all-cause mortality was observed in 8 patients (29%). We performed a Kaplan–Meier analysis to evaluate time to the first occurrence of the composite endpoint during follow-up. This analysis revealed a statistically significant difference (log-rank test, *p* = 0.007) between patients with residual PVL < moderate and those with residual PVL ≥ moderate (Fig. 4). Patients with residual PVL ≥ moderate demonstrated a significantly shorter time to the composite endpoint, indicating a higher risk of adverse events during the two-year follow-up period. PVL severity was classified based on intra-procedural TEE findings at the end of the procedure.

### Echocardiographic changes

**Fig. 5:**
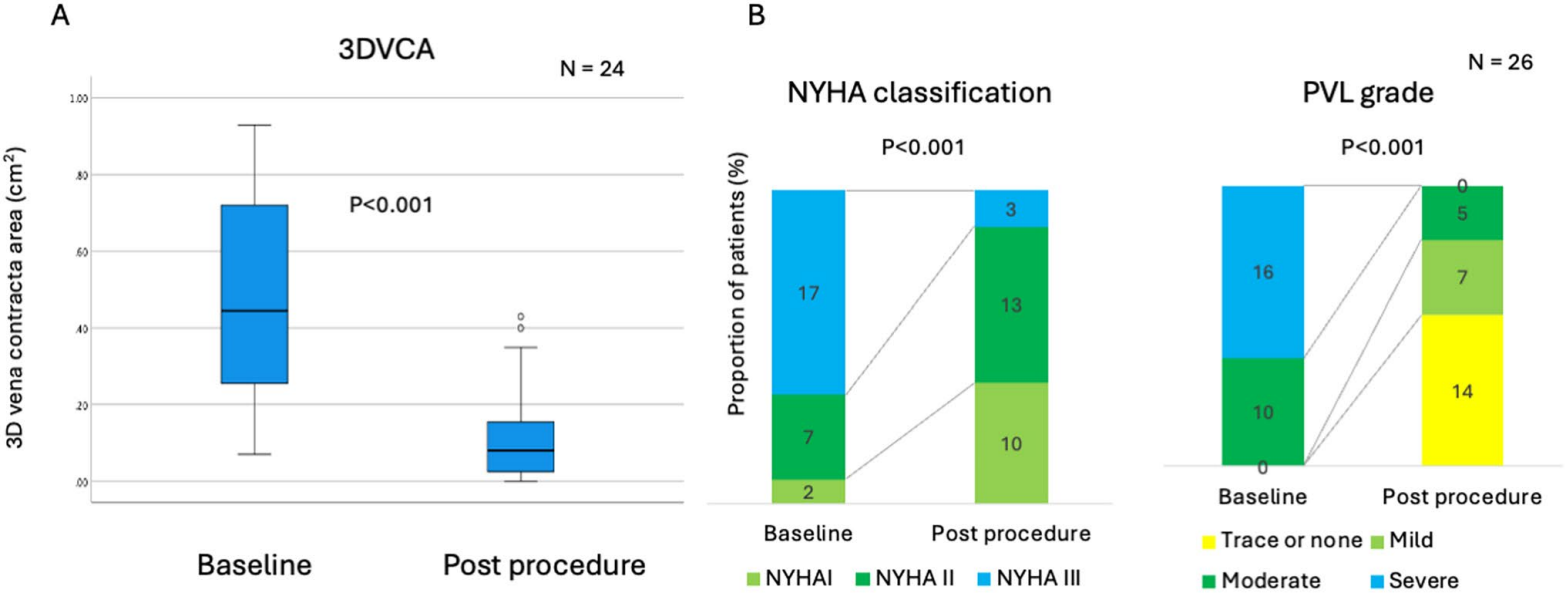
Echocardiographic and clinical improvement after transcatheter paravalvular leak (PVL) closure. **A**: The change in the 3DVCA assessed by 3D TEE (*n* = 24). **B**: The change in the NYHA classification and PVL grade from baseline to post-procedure (*n* = 26, excluding two cases requiring emergency surgery). The y-axis represents the proportion of patients (%), and numbers within bars indicate the actual number of patients in each category. Both NYHA class and PVL grade showed significant improvement after PVL closure (*p* < 0.001). 3DVCA, three-dimensional vena contracta area; TEE, transesophageal echocardiography; NYHA, New York Heart Association Functional Classification

Echocardiographic data before and after PVL closure were available for 26 of the 28 enrolled patients (22 mitral and 4 aortic). Two patients (one mitral and one aortic) who required emergent surgical conversion during the procedure were excluded from this comparison. Quantitative assessment of the 3DVCA was feasible in 24 patients (including both mitral and aortic positions). We compared the pre- and post-intervention echocardiographic parameters. Significant reductions (*p* < 0.001) were observed in the mitral valve maximum VC width (a measure of regurgitant orifice size), color Doppler flow jet area (representing regurgitant volume), and 3DVCA (a measure of regurgitant severity) in the mitral valve position. Specifically, the maximum VC width decreased from 10.6 (IQR 8.0–13.5) mm to 2.6 (IQR 1.2–3.8) mm, the color flow jet area decreased from 4.8 (IQR 2.4–5.5) cm² to 0.7 (IQR 0.3–2.5) cm², and the 3DVCA decreased from 0.45 (IQR 0.24–0.76) cm² to 0.1 (IQR 0.02–0.18) cm². In the aortic valve position, we observed non-significant trends towards a reduction in the maximum VC width (*p* = 0.11) and 3DVCA (*p* = 0.07) (Table 3; Fig. 5a).

**Table 3:** Echo parameters baseline and post closure in patients with paravalvular leak

Aortic position *N* = 4 (Excluding cases requiring emergency surgery)	Baseline	Post PVL closure (intra-procedural)	*P* value
**PVL severity**			
Trivial or mild	0	3 (75)	0.1
Mild to moderate	0	1 (25)	
Moderate	2 (50)	0	
Severe	2 (50)	0	
**Transesophageal echocardiography**			
Descending aorta flow			0.046
Absent	0	4 (100)	
Absent or brief early diastolic	0	0	
Intermediate	4 (100)	0	
Prominent, holo-diastolic	0	0	
Proximal flow convergence Absent Possible Often present	0 1 (25) 3 (75)	4 (100) 0 0	0.06
Maximum VC width (2D or 3D), mm	10.6 (8.5, 15.3)	2.4 (0.8, 2.9)	0.11
VCA (2D or 3D), cm^2^	0.45 (0.30, 0.55)	0.06 (0.04, 0.09)	0.07
Circumferential extent, %	22 (22, 30)	5 (5, 5)	0.07
**Mitral position** *N* = 22 (Excluding cases requiring emergency surgery)	**Baseline**	**Post PVL closure (intra-procedural)**	**P value**
**PVL severity**			
Trivial or mild	0	11 (50)	*P* < 0.001
Mild to moderate	0	6 (27)	
Moderate	8 (36)	5 (23)	
Severe	14 (64)	0	
**Transesophageal echocardiography**			
Maximum VC width (2D or 3D), mm	10.6 (8.0, 13.5)	2.6 (1.2, 3.8)	< 0.001
VCA (2D or 3D), cm^2^	0.45 (0.24, 0.76)	0.1 (0.02, 0.18)	< 0.001
Color flow jet area, cm^2^	4.8 (2.4, 5.5)	0.7 (0.3, 2.5)	< 0.001
**Transthoracic echocardiography**			
*N* = 26 (overall cohort including both mitral and aortic PVL; excluding cases requiring emergency surgery)	**Baseline**	**Post PVL closure (at discharge)**	**P value**
LVDd, mm	52 (48, 58)	51 (48, 61)	0.86
LVDs, mm	33 (31, 43)	37 (32, 45)	0.24
LVEDV, ml	132 (107, 171)	124 (110, 185)	0.91
LVESV, ml	45 (39, 86)	55 (38, 92)	0.2
LVEF, %	60 (46, 64)	54 (47, 62)	0.1
LAD, mm	61 (52, 70)	60 (50, 72)	0.75
TRPG, mmHg	32 (28, 44)	30 (22, 36)	0.009
Estimated RA pressure, mmHg	8 (3, 15)	8 (3, 15)	0.37

Two patients (one mitral and one aortic) who required emergent surgical conversion were excluded from echocardiographic comparison

Values are expressed as median [interquartile range (IQR)] unless otherwise indicated. Categorical variables are presented as number (percentage)

TTE parameters were analyzed in the overall cohort (*N* = 26) combining mitral (*n* = 22) and aortic (*n* = 4) PVL cases

PVL, paravalvular leakage; VC, venacontracta; 2D, two dimensional; 3D, three dimensional; LVDd, left ventricular end-diastolic dimension; LVDs, left ventricular end-systolic dimension; LVEDV, left ventricular end-diastolic volume; LVESV, left ventricular end-systolic volume; LVEF, left ventricular ejection fraction; LAD, left atrial dimension; TRPG, trans tricuspid pressure gradient; RA, right atrial

### Functional improvement

The percutaneous PVL closure demonstrated a significant positive impact on both the patients’ symptoms and the severity of PVL. A substantial number of patients experienced an improvement in their NYHA functional class post-procedure (*p* < 0.001), indicating a clinically relevant reduction in HF symptoms within a relatively short hospital stay (median 8 [7–12] days overall, as shown in Table 2). Furthermore, a significant reduction in PVL severity was achieved (*p* < 0.001), accompanied by a notable decrease in the prevalence of moderate and severe PVL. Combined, these improvements underscore the effectiveness of this interventional approach in enhancing quality of life for patients with symptomatic PVL (Fig. 5b).

## Discussion

### Summary of key findings

This single-center study demonstrated the safety and effectiveness of percutaneous PVL closure as a therapeutic option for patients with paravalvular regurgitation following surgical valve replacement. During a median follow-up of 2 years, all-cause mortality was 29%, and the composite endpoint occurred in 64% of patients. The procedural success rate was 71%, with significant improvement in echocardiographic and hemolysis parameters, establishing both procedural feasibility and clinical benefit in this high-risk population (Tables 3 and 4) [16, 17].

**Table 4:** Laboratory data baseline and post closure in patients with paravalvular leak

*N* = 26 (Excluding cases requiring emergency surgery)	Baseline	Post PVL closure (at discharge)	*P* value
LDH, U/L	1888 (1101, 3694)	1392 (903, 2465)	0.003
Total bilirubin, mg/dl	2.2 (1.6, 3.5)	1.6 (1.2, 2.2)	0.011
NT-pro BNP, pg/ml	2646 (1735, 4042)	1373 (713, 3149)	0.14
Hb, g/dl	9.4 (9.0, 10.4)	9.4 (8.6, 10.6)	0.67
Creatinine, mg/dl	1.2 (0.9, 1.7)	1.2 (0.9, 1.6)	0.81
ALB, g/dl	3.8 (3.7, 4.2)	3.6 (3.2, 4.2)	0.043
CRP, mg/dl	0.1 (0.1, 0.8)	0.5 (0.1, 1.4)	0.17

Values are shown as median [Q1, Q3]

PVL, paravalvular leakage; LDH, lactate dehydrogenase; NT-proBNP, N-terminal pro-brain natriuretic peptide; Hb, hemoglobin; ALB, albumin; CRP, C reactive protein

The referring Heart Team considered redo surgery to carry an unacceptably high risk in most patients because of advanced heart failure, renal dysfunction, hemolysis, and prior sternotomies. Consequently, transcatheter PVL closure was selected as the preferred strategy.

Two serious intraoperative complications occurred, both of which required emergency surgery: one involving mitral annular dehiscence and another left ventricular perforation during sheath insertion. These events emphasize the technical complexity of PVL closure and the importance of experienced Heart Team involvement.

Left ventricular perforation during PVL closure may occur during advancement or manipulation of relatively stiff delivery sheaths across angulated or fragile ventricular myocardium, particularly in patients with prior surgery and altered ventricular geometry. Annular dehiscence may be precipitated by excessive traction or device oversizing at a structurally weakened annulus, especially in the setting of severe calcification or prior surgical disruption. These mechanisms highlight the importance of meticulous imaging guidance, cautious catheter manipulation, and appropriate device selection in complex PVL anatomy.

Reoperative surgery for PVL is associated with unique anatomical and biological challenges that substantially increase procedural risk. Dense postoperative adhesions make re-entry technically demanding and raise the risk of cardiac or great vessel injury. Moreover, PVL frequently arises from fragile or previously infected annular tissue, which predisposes to recurrent leakage even after surgical re-repair. Importantly, these PVL-specific factors are not fully reflected in conventional surgical risk models such as the STS score, suggesting that the true operative risk is often considerably higher than predicted.

Consequently, the multidisciplinary heart team consistently judged transcatheter PVL closure to be the most appropriate therapeutic strategy for this population.

The use of dedicated PLD occluders may contribute to improved procedural efficiency and radiation exposure. These findings are consistent with previous reports demonstrating favorable technical outcomes with purpose-designed PLD devices in complex anatomy [16, 18].

### Comparison with previous studies

Despite differences in device availability and patient risk profiles, the procedural success rate observed in this study (71%) is comparable to that reported in major international registries, such as Ruiz et al. (76%) and Calvert et al. (77%) [8, 18]. While European and North American series frequently report success rates exceeding 85–90%, these studies often include centers with access to dedicated devices such as the AVP III or PLD. In contrast, our earlier experience relied primarily on AVP II, which is not optimized for PVL closure, thereby partly explaining the slightly lower success rate. The procedural success rate in this study (71%) was modest compared with international series (> 85%), reflecting Japan-specific limitations and case complexity. During most of the study period, the AVP III device was not available in Japan. Notably, following completion of the RESEAL trial, the dedicated PLD received regulatory approval in Japan in 2023, representing a significant step forward in expanding therapeutic options for transcatheter PVL closure in this region. Moreover, our institution performed the first percutaneous PVL closure in Japan in 2014 [19] and has accumulated experience since then, indicating that early learning-phase cases were included in this cohort. Part of our earlier experience was included in the initial report by Niikura et al., which described 12 cases from our institution in which AVP II was used [9]. The present study expands upon that series to include all subsequent patients and devices, representing the largest single-center experience of transcatheter PVL closure in Japan to date. Consistent with the observations of Sorajja et al. [7], who demonstrated a clear learning curve effect in percutaneous PVL closure, our data also suggest that procedural outcomes can improve with accumulating operator experience and systematic Heart Team collaboration. These findings underscore the significance of specialized expertise and institutional continuity in achieving consistent procedural success in Japan.

Notably, the first case included in this series, performed in 2014, represented the first percutaneous PVL closure ever conducted in Japan [19].

### Imaging and procedural insights

Intraoperative TEE, particularly 3D imaging, was indispensable for accurately characterizing leaks and sizing devices, as well as providing real-time procedural guidance. Quantitative 3D parameters, such as VCA, provided reproducible intraoperative endpoints and were closely associated with procedural success (Fig. 2). The integration of multimodal imaging, including CT for preprocedural planning and 3D TEE for guidance, was key to minimizing complications such as device interference and residual regurgitation. These results reinforce the importance of developing an imaging-based strategy to optimize outcomes in complex PVL cases (Fig. 5a, b).

Due to its availability and ease of use, the AVP II was the most frequently used device in our series; however, its circular configuration occasionally caused suboptimal sealing or interfered with mechanical valve leaflets. The later introduction of the dedicated PLD, with its rectangular and square design, facilitated better adaptation to irregular PVL shapes, often reducing the number of devices required per case. The AVP III, which features an oval geometry and is widely used in the United States, offers superior conformity to PVL morphology; however, it remains unavailable in Japan.

### Clinical implications

Surgical reoperation, including minimally invasive cardiac surgery (MICS), remains the definitive treatment option for selected patients. However, conducting redo surgery following valve replacement is technically demanding due to postoperative adhesions, distorted anatomy, and the potential risk of cardiac or prosthetic injury.

Conversely, for patients with high or prohibitive surgical risk, transcatheter PVL closure offers a feasible and less invasive alternative that can achieve symptom relief and hemodynamic improvement when anatomical conditions are suitable for device implantation.

Consistent with previous international reports, our Kaplan–Meier analysis (Fig. 4) showed that patients with a residual PVL < moderate exhibited significantly better event-free survival (*p* = 0.007). These findings reinforce the strong association between success and long-term prognosis, indicating that achieving residual PVL < moderate is a key determinant of favorable outcomes. This finding also aligns with the multicenter report by Wells et al. [20], which demonstrated that a residual PVL ≥ moderate was an independent predictor of mortality and HF hospitalization following percutaneous closure. Together, these data highlight the prognostic importance of achieving near-complete leak elimination whenever technically feasible.

These results emphasize the clinical importance of meticulous imaging-based planning and procedural optimization in minimizing residual PVL, as even mild residual regurgitation can adversely affect long-term prognosis. Future studies comparing catheter-based and contemporary surgical or MICS approaches could further refine patient selection and help define the complementary roles of each strategy in clinical practice.

### Limitations and future directions

This study has several limitations. As a retrospective, single-center analysis with a small sample size, the generalizability of its findings is limited.

The follow-up period was relatively short, and long-term outcomes—including late hemolysis and device durability—are yet to be clarified. Future multicenter, prospective registries with standardized imaging and outcome measures are warranted to corroborate these findings and evaluate newer PVL-specific devices as they become available in Japan.

## Conclusions

Percutaneous PVL closure demonstrated acceptable procedural success and meaningful clinical improvement in a high-risk surgical population. Statistically significant improvements were observed in both echocardiographic and clinical outcomes, suggesting it confers a potential benefit in reducing symptoms and improving hemodynamic function. However, the association between PVL severity and adverse events highlights the importance of comprehensive preprocedural evaluation and individualized planning. Future large-scale, prospective studies will be crucial for refining patient selection and establishing standardized treatment algorithms.

## Supplementary Information

Below is the link to the electronic supplementary material.

Supplementary Material 1

Supplementary Material 2

Supplementary Material 3

## Data Availability

The deidentified participant data that support the findings of this study, including clinical characteristics, procedural details, and echocardiographic measurements, will be available from the corresponding author upon reasonable request. The study protocol and statistical analysis plan are also available. Data will be made available beginning 3 months after publication and for up to 5 years, to researchers who provide a methodologically sound proposal for use in achieving the aims of the approved proposal. Requests should be directed to gou.hashimoto@med.toho-u.ac.jp.
