# Supplementary material for: Percutaneous paravalvular leak closure: clinical outcomes and practical insights from a single-center experience in Japan
Source: Cardiovasc Interv Ther. 2026 Apr 24;41(3):737–48. doi: 10.1007/s12928-026-01273-3 (PMC13279666; doi:10.1007/s12928-026-01273-3)
Supplement: Supplementary file 1 — Supplementary Material 1 [file 12928_2026_1273_MOESM1_ESM.docx]

**Mitral PVL severity**

|  | **Trivial** | **Mild** | **Moderate** | **Severe** |
| --- | --- | --- | --- | --- |
| **PV** | Systolic dominance | Systolic dominance | Systolic blunting | Systolic flow reversal |
| **Proximal flow convergence** | Absent | Minimal | Intermediate | Large |
| **VC width, mm** | <1.5 | 1.5–2.9 | 3.0–6.9 | ≧7.0 |
| **VCA, cm^2^** | <0.05 | 0.05–0.19 | 0.2–0.39 | ≧0.4 |
| **Color flow jet area, cm^2^** | <2 | 2-3.9 | 4-9.9 | ≧10 |

**Aortic PVL severity**

|  | **Trivial** | **Mild** | **Moderate** | **Severe** |
| --- | --- | --- | --- | --- |
| **Descending aorta flow** | Absent | Absent or brief early diastolic | Intermediate | Prominent, holo-diastolic |
| **Proximal flow convergence** | Absent | Absent | Possible | Often present |
| **VC width, mm** | <1.5 | 1.5–2.9 | 3.0–6.9 | ≧7.0 |
| **VCA, cm^2^** | <0.05 | 0.05-0.19 | 0.2-0.39 | ≧0.4 |
| **Circumferential extent, degree** | <5 | 5–9 | 9–29 | ≧30 |
