# Supplementary material for: Percutaneous paravalvular leak closure: clinical outcomes and practical insights from a single-center experience in Japan
Source: Cardiovasc Interv Ther. 2026 Apr 24;41(3):737–48. doi: 10.1007/s12928-026-01273-3 (PMC13279666; doi:10.1007/s12928-026-01273-3)
Supplement: Supplementary file 2 — Supplementary Material 2 [file 12928_2026_1273_MOESM2_ESM.docx]

**Supplementary Table 1. Procedural metrics by device type**

| **Parameter** | **AVP II / ADO II (n = 19)** | **PLD (n = 8)** | ***p*-value** |
| --- | --- | --- | --- |
| **Procedure time (min)** | 281 (239, 408) | 254 (156, 316) | 0.24 |
| **Number of devices per case** | 3.0 (2.0, 3.5) | 1.0 (1.0, 1.2) | <0.001 |
| **Fluoroscopy time (min)** | 158 (110, 242) | 116 (74, 140) | 0.22 |
| **Radiation dose (mGy)** | 2781 (1527, 3532) | 1397 (1190, 1848) | 0.034 |

Comparisons were made between procedures performed using AVP II/ADO II (n = 19) and those using the dedicated Occlutech Paravalvular Leak Device (PLD) (n = 8), after excluding one case in which both devices were used.

Data are expressed as median [interquartile range] or number (%), as appropriate.

Procedures performed with the dedicated PLD required significantly fewer devices per case and were associated with lower radiation exposure, reflecting improved procedural efficiency in the later phase of experience.

Abbreviations: AVP, Amplatzer Vascular Plug; ADO, Amplatzer Duct Occluder; PLD, Paravalvular Leak Device; IQR, interquartile range; DAP, dose–area product; PSD, peak skin dose.
