# Supplementary figures and images for: Percutaneous paravalvular leak closure: clinical outcomes and practical insights from a single-center experience in Japan
Source: Cardiovasc Interv Ther. 2026 Apr 24;41(3):737–48. doi: 10.1007/s12928-026-01273-3 (PMC13279666; doi:10.1007/s12928-026-01273-3)

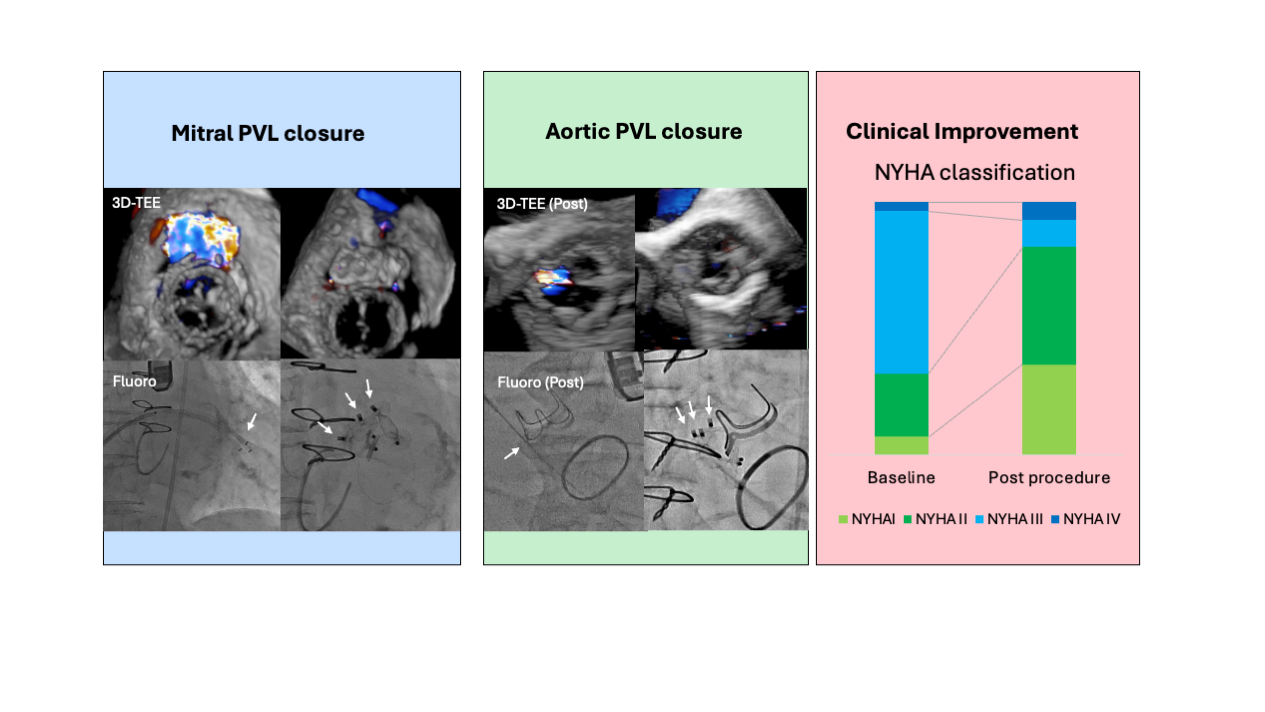

Supplement: Supplementary file 3 — Supplementary Material 3 [file 12928_2026_1273_MOESM3_ESM.tiff]
